# Electroconvulsive therapy improves somatic symptoms before mood in patients with depression: a directed network approach

**DOI:** 10.1192/j.eurpsy.2024.71

**Published:** 2024-08-27

**Authors:** K. Hebbrecht

**Affiliations:** Psychiatry, University Psychiatric Hospital KULeuven, Leuven, Belgium

## Abstract

**Abstract:**

The recent network perspective of depression conceptualizes depression as a dynamic network of causally related symptoms, this in contrast with the traditional view of depression as a discrete latent entity that causes all symptoms. Electroconvulsive therapy (ECT) is an effective treatment for severe depression, but little is known about the temporal trajectories of symptom improvement during a course of ECT. We will present the results of a study that investigates the temporal trajectories of individual symptoms during treatment with ECT.

**Disclosure of Interest:**

None Declared

